# Platelet Rich Concentrate Promotes Early Cellular Proliferation and Multiple Lineage Differentiation of Human Mesenchymal Stromal Cells *In Vitro*

**DOI:** 10.1155/2014/845293

**Published:** 2014-11-10

**Authors:** Samuel Shani, Raja Elina Ahmad, Sangeetha Vasudevaraj Naveen, Malliga Raman Murali, Karunanithi Puvanan, Azlina Amir Abbas, Tunku Kamarul

**Affiliations:** ^1^Department of Physiology, Faculty of Medicine, University of Malaya, Lembah Pantai, 50603 Kuala Lumpur, Malaysia; ^2^Tissue Engineering Group (TEG), Department of Orthopaedic Surgery (NOCERAL), Faculty of Medicine, University of Malaya, Lembah Pantai, 50603 Kuala Lumpur, Malaysia; ^3^Clinical Investigative Centre (CIC), University Malaya Medical Centre, Kuala Lumpur, Malaysia

## Abstract

Platelet rich concentrate (PRC) is a natural adjuvant that aids in human mesenchymal stromal cell (hMSC) proliferation *in vitro*; however, its role requires further exploration. This study was conducted to determine the optimal concentration of PRC required for achieving the maximal proliferation, and the need for activating the platelets to achieve this effect, and if PRC could independently induce early differentiation of hMSC. The gene expression of markers for osteocytes (ALP, RUNX2), chondrocytes (SOX9, COL2A1), and adipocytes (PPAR-*γ*) was determined at each time point in hMSC treated with 15% activated and nonactivated PRC since maximal proliferative effect was achieved at this concentration. The isolated PRC had approximately fourfold higher platelet count than whole blood. There was no significant difference in hMSC proliferation between the activated and nonactivated PRC. Only RUNX2 and SOX9 genes were upregulated throughout the 8 days. However, protein expression study showed formation of oil globules from day 4, significant increase in ALP at days 6 and 8 (*P* ≤ 0.05), and increased glycosaminoglycan levels at all time points (*P* < 0.05), suggesting the early differentiation of hMSC into osteogenic and adipogenic lineages. This study demonstrates that the use of PRC increased hMSC proliferation and induced early differentiation of hMSC into multiple mesenchymal lineages, without preactivation or addition of differentiation medium.

## 1. Introduction

The current resurgence of interest in the field of tissue engineering and regenerative medicine has driven many researchers to explore the potential use of cell-based therapy, especially that involving the use of adult mesenchymal stem or stromal cells (MSCs). MSCs have the ability to self-renew, modulate immune responses, and exhibit multilineage differentiation potential. Due to these distinct characteristics, the potentials of MSCs in clinical applications have been widely speculated, and in many studies, the use of these multipotent cells has demonstrated good outcomes. However, in order for MSC treatment to be effective, the cells need to be of sufficient numbers, usually in the order of millions, in addition to having the ability to undergo directed lineage differentiation. Thus, it has become apparent that the use of adjuncts that can dramatically improve the proliferation and differentiation of the MSCs* in vitro* is of paramount importance. Of the many known biological products that has been previously described, platelet rich concentrate (PRC), that is, enriched levels of platelets relative to whole blood [1], has recently emerged as a potential tool that may result in these desirable outcomes [2]. The regenerative property of the platelets is reckoned to be the result of the release of various growth factors, cytokines, and chemokines as the platelets are activated after being exposed to certain factors such as thrombin. PRC has thus increasingly been incorporated into clinical practice, such as in plastic surgery [3, 4] and orthopaedics [5–12]. However, despite being a therapeutic tool that is recognized for promoting tissue regeneration, the actual mechanism involved is still not well understood. It has been previously assumed that PRC acts by promoting cell proliferation [13–17]. Furthermore, it has been suggested that by using PRC on cells of relatively high potency like progenitor cells and MSCs, cellular differentiation can be expected [1, 5, 7, 17]. It is therefore a strong possibility that the regenerative effects observed in clinical applications of PRC may have been due to its effect in augmenting both proliferation and differentiation of multipotent cells. Unfortunately, studies reporting the synergistic effect of PRC in enhancing the proliferative and differentiation potential of MSCs appear to be limited. In addition, previous studies have not come to the consensus on the optimal concentration of PRC that is required to be effective on MSCs and whether activation of PRC would be required in order to produce the desired effects. The present study was therefore conducted to determine the optimal concentration of activated and nonactivated platelet rich concentrate (PRC) required for optimal proliferation of the bone marrow-derived human mesenchymal stromal cells (hMSCs)* in vitro*, to establish the need for PRC activation prior to its* in vitro* use to achieve this and to determine the effect of PRC on their differentiation potential, thereby demonstrating the potential of PRC for MSC-based therapies.

## 2. Materials and Methods

### 2.1. Preparation of PRC

The protocol for this study was approved by University of Malaya Medical Centre Medical Ethics Committee (UMMC reference number 967.10). Blood (25 mL) was collected from six healthy volunteers after obtaining a written informed consent. Blood samples were transferred into vacutainers containing ACD-A anticoagulant. PRC was prepared using double centrifugation method as described earlier with slight modifications [18, 19]. Briefly, the anticoagulated blood was centrifuged at 1500 rpm for 10 minutes to initially separate the various components of blood. To minimise interindividual variations, the plasma and buffy coat containing platelets that were isolated were pooled in a 50 mL falcon tube and centrifuged again at 3000 rpm for 10 minutes. The supernatant portion of the plasma was discarded and only the platelet pellets were isolated and resuspended in sterile phosphate buffered saline (PBS, pH 7.2), at 1/10th the initial blood volume [20]. This constitutes the platelet-rich concentrate (PRC). Prostacyclin (PGI2) (0.5 *μ*L in 1 mL of platelet suspension) was added to prevent the transitory activation of platelets during the centrifugation and resuspension steps [21]. The amount of platelets in PRC and whole blood were then determined using Sysmex XE 5000 hematology analyser. The concentration of platelets in the PRC isolated each time ranged from 1100 to 1200 × 10^3^/*μ*L, which was at least 3-fold more than that of the normal plasma reference range. The PRC was then divided into two equal portions. One portion was activated using 10% calcium chloride and lyophilised human thrombin (1 : 1 (v/v)). The activator was added at a ratio of 1 : 10 (v/v) to one portion of the PRC and, following that, the tubes were incubated at room temperature. After a firm clot has been obtained, the tubes were centrifuged at 3200 rpm for 5 minutes [22]. The supernatant that presumably contains most of the platelet contents was obtained and referred to as the activated PRC. The other portion of platelet suspension (i.e., whole platelet pellets in PBS) was used directly without any activation and is referred to as the nonactivated PRC. It is presumed that the platelet contents in the nonactivated PRC remained in the intact structure of the pellets. Thus, the whole suspension was used. The effect of a range of concentrations, that is, 5%, 10%, 15%, 20%, and 25% (v/v), of both activated and nonactivated PRC on hMSC proliferation was subsequently tested.

### 2.2. Scanning Electron Microscopy

SEM was done as a qualitative assessment of platelet activation and to visualize the morphological features of activated and nonactivated platelets. The platelets were fixed overnight in 4% glutaraldehyde in 0.1 M cacodylate and postfixed for 1 hour in 1% osmium tetroxide. It was then washed three times in distilled water before being dehydrated through a graded ethanol series (50, 75, 95, and 100%). Hexamethyldisilazane (HMDS) was added to the fixed platelets for 10 minutes and then it was left to dry in a desiccator. The dried specimens were mounted on aluminium stubs with adhesive carbon tapes and sputter-coated with gold before being examined using a digital scanning electron microscope.

### 2.3. Flow Cytometry Analyses of Platelet Quality

The activation of platelet during the PRC preparation process was determined by the expression of CD62 on the surface of the outer membrane of platelet using flow cytometer (Becton Dickinson, San Jose, CA). Anti-CD61-PerCP, anti-CD41a-APC, and PE-anti-P-selectin (CD62P) (Becton Dickinson) (2 *μ*L each) were added to 5 *μ*L of nonactivated PRC. The mixtures were incubated in the dark for 20 min at room temperature, after which the platelets were fixed by addition of 1 mL of cold (2° to 8°C) 1% paraformaldehyde and analysed after 30 minutes incubation in the dark. Forward scatter (FSC), side scatters (SSC), and fluorescence data were obtained with gain settings in the logarithmic mode. Platelets were selected based on their platelet marker positivity and FSC/SSC characteristics.

### 2.4. Growth Factor Quantification

Concentrations of growth factors PDGF-AA, PDGF-BB, PDGF-AB, TGF-*β*1, VEGF, FGF-2, and IGF-1 were determined in the activated and nonactivated PRC and whole blood using the commercially available Enzyme Linked Immunosorbent Assay (ELISA) kits according to the manufacturer's instructions.

### 2.5. Human Mesenchymal Stromal Cell (hMSC) Isolation

Bone marrow was aspirated from six patients undergoing total knee/hip arthroplasty in the University of Malaya Medical Centre with approval from the Medical Ethics Committee of the institution (UMMC, reference number 967.10). Written informed consent was obtained from each patient. Aspirated bone marrow was added to equal volume of phosphate-buffered saline (PBS; pH 7.2), layered onto Ficoll-Paque Premium of density 1.073 g/mL (GE Healthcare, Sweden), and centrifuged at 2,200 rpm for 25 minutes. The mononuclear cells were then isolated and resuspended in 10 mL of low glucose Dulbecco's modified eagle medium (L-DMEM) and centrifuged again at 1800 rpm for 5 minutes. The supernatant was discarded and the cell pellet obtained was cultured in growth medium (L-DMEM supplemented with 10% fetal bovine serum (FBS), 1% Penicillin/Streptomycin (100 U/mL, Invitrogen-Gibco), and 1% Glutamax-1 (Invitrogen-Gibco, USA)) in T-25 tissue culture flasks. Medium was changed every 3 days until the cultures were 80% confluent. The cells were then serially passaged, and passage 2 cells were used for further experiments.

### 2.6. *In Vitro* Cell Proliferation Assay

Human MSCs were seeded in 24-well culture plates at a density of 1.5 × 10^3^ cells/well. After 48 hours of culture in growth medium, the cells were subjected to a serum reduction of 1% FBS to arrest cell cycle progression for 24 hours. The normal growth medium was then supplemented with different concentrations of activated and nonactivated PRC (5%, 10% 15%, 20%, and 25% v/v) in each well except for the control wells. Cell viability was observed at 0, 2, 4, 6, and 8 days using alamarBlue assay kit according to the manufacturer's protocol. Standard curve for total number of hMSCs versus absorbance was plotted (data not shown) and used to extrapolate the cell numbers of the treated and the control samples. All experiments were performed in triplicate and repeated six times.

### 2.7. Gene Expression

The expression of osteocytes (ALP, RUNX2), chondrocytes (SOX9, COL2A1), and adipocytes (PPAR-*γ*) markers was analyzed using the QuantiGenePlex 2.0 (set 12216) Assay Kit (Panomics/Affymetrix, Inc., Fremont, CA). The cells treated with 15% activated and nonactivated PRC were trypsinized after each time point and lysed to release the RNAs and were then incubated overnight with target specific probe sets. Beads and bound target RNA were then washed and sequentially hybridized with preamplifier, amplifier, and label probe (biotin) and incubated with streptavidin-conjugated R-phycoerythrin (SAPE), which binds to the biotinylated probes. The resulting fluorescence signal associated with individual capture beads was read on a Luminex flow cytometer. The signal, reported as median fluorescence intensity (MFI), is proportional to the number of target RNA molecules present in the sample. Signals from three housekeeping genes, namely, hypoxanthine-guanine phosphoribosyltransferase 1 (HPRT1), phosphoglycerate kinase 1 (PGK1), and TATA-box binding protein (TBP), were used to normalize the gene expression data for the test and control samples.

### 2.8. *In Vitro* Differentiation Assays and Histological Analysis

Assays were performed in culture supernatants and in triplicate at 2, 4, 6, and 8 days. Lipid droplets within the cells were stained with Oil Red O dye using the adipogenesis assay kit (Cayman Chemical, Ann Arbor, MI) according to the manufacturer's instructions and intracellular lipid accumulation was quantified by the elution of Oil Red O from the lipid droplets by adding dye extraction solution for 10 minutes and the optical density (OD) was measured at 490 nm. Alkaline phosphatase assay kit (BioVision, CA, USA) was used to measure the ALP activity according to the manufacturer's instructions. ALP assay involves dephosphorylation of pNPP (p-nitrophenyl phosphate) by ALP to a yellow colored pNP (p-nitrophenol), the absorbance of which is measured at 405 nm. Later, the cells were fixed in formalin, washed, and stained with Alizarin red S to detect the formation of mineral nodules. Glycosaminoglycan amount was measured using the Blyscan assay kit (Biocolor Ltd., UK). Briefly, Blyscan dye reagent (500 *μ*L) was added to the supernatants (500 *μ*L) and the kit standard and mixed for 30 minutes at room temperature. Sulfated GAG was precipitated by centrifugation at 12,000 rpm for 10 minutes. Bound dye was released with the dissociation reagent (500 *μ*L) and the absorbance was measured using a spectrophotometer at 656 nm for GAG [13] and 750 nm for protein. GAG content was normalized with the total protein content (GAGs *μ*g/protein mg). The cells were then fixed, washed, and stained with Safranin O to detect the release of GAG. Cells treated with the commercially available adipogenic osteogenic and chondrogenic differentiation medium (Invitrogen-Gibco, USA) were used as the positive control for the histological staining.

### 2.9. Statistical Analysis

All values are expressed as mean ± standard deviation. The differences between groups were analysed using a nonparametric test (Kruskal-Wallis). If values were significant, Mann-Whitney *U* tests were performed to evaluate the level of significance between the groups. Differences were considered to be significant at *P* ≤ 0.05. Data were analysed with SPSS software version 17.0 (IBM Corp., Armonk, NY, USA).

## 3. Results

### 3.1. Platelet Count

The PRC preparation had significantly higher platelet count (1140 ± 98.85 × 10^3^ platelets/*μ*L), with an average of approximately fourfold increase compared to the whole blood (251.71 ± 27.93 × 10^3^ platelets/*μ*L) (*P* = 0.021).

### 3.2. Scanning Electron Microscopy

**Figure 1:**
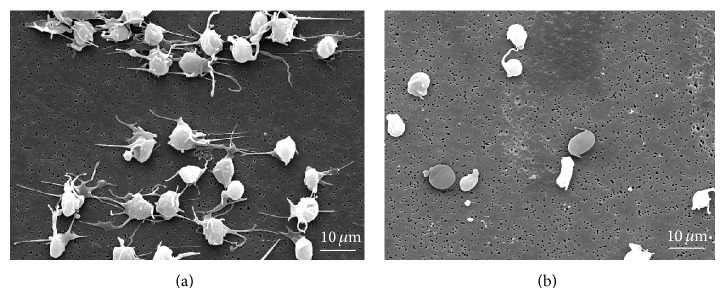
Scanning Electron Microscopy showing activated (a) and nonactivated (b) platelets in PRC.

Nonactivated platelets (Figure 1(a)) appear as oval disks while the activated platelets (Figure 1(b)) change shape and form pseudopodia, which help in the release of their granular contents, aggregation and adherence to the damaged tissue, and ultimately clot formation.

### 3.3. Flow Cytometric Analyses

**Figure 2:**
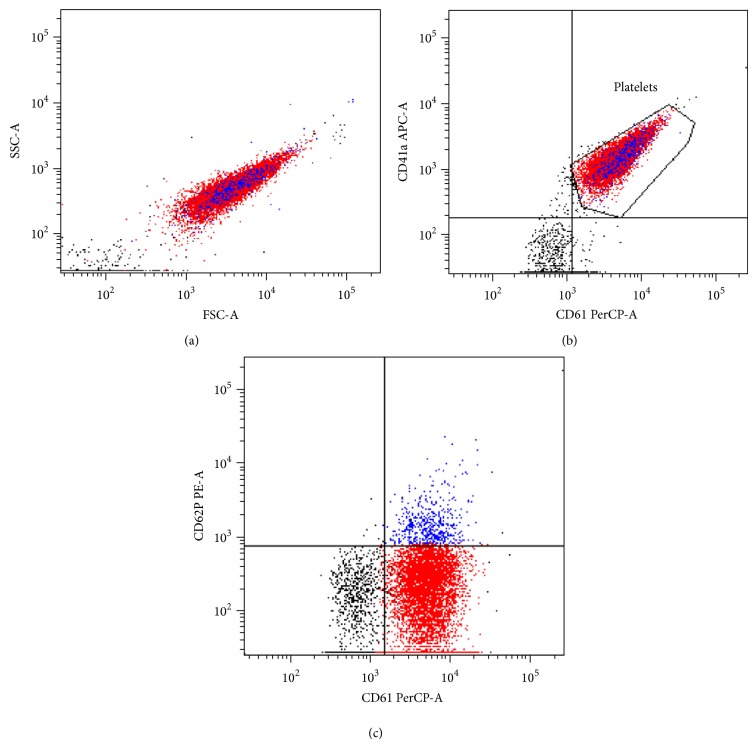
PRC sample is gated based on size (forward scatter) and granularity (side scatter) (a). CD61^+^ versus CD41^+^ includes only the population of platelets positive to both the markers (b). CD61^+^ versus CD62^+^ shows the platelets in the nonactivated PRC positive to CD61^+^ and about 5.2% positive to CD62^+^ (c).

The region corresponding to the platelet population was delimited according to their size and granularity (Figure 2(a)). Both CD41^+^ and CD61^+^, present normally on the surface of the platelets, were expressed in 86.9% of the selected cells, confirming that the population included was essentially composed of platelets (Figure 2(b)). Only about 5.2% of the cells in the nonactivated sample expressed CD62^+^ (Figure 2(c)), a CD marker that is uniquely expressed on activated platelets. This signifies that the platelets in the nonactivated PRC were not subjected to any inadvertent activation during the preparation process.

### 3.4. Growth Factor Quantification

**Figure 3:**
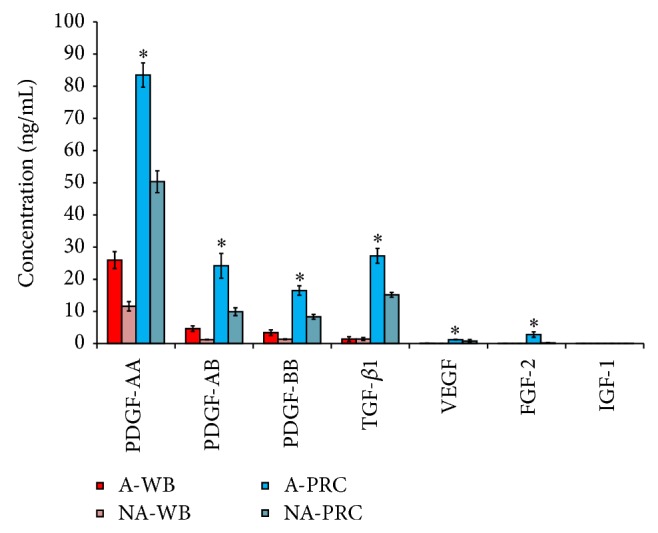
Concentration of various growth factors released in whole blood and PRC (before and after activation). The growth factors were significantly higher in activated PRC compared to activated whole blood (^*^ *P* ≤ 0.05).

Growth factors could be detected in all of the samples. In general, the measured growth factors concentrations were higher in the activated PRC compared to the nonactivated counterparts. The level of PDGF-AA in the activated PRC (83.48 ± 3.77 ng/mL) was higher compared to all other growth factors, with a threefold increased concentration compared to that found in the activated whole blood (25.969 ± 2.61 ng/mL). Concentration of IGF in the activated PRC was 0.036 ± 0.009 ng/mL, which was very low compared to the concentration of other growth factors. Activated PRC showed a significant increase in the concentration of all growth factors compared to the nonactivated PRC. Similarly, the growth factors concentrations in the activated whole blood were significantly higher than the nonactivated whole blood (*P* ≤ 0.05). In addition, a significant difference was observed between activated PRC and activated whole blood for all growth factors except IGF-1 (*P* = 0.189) (Figure 3).

### 3.5. Effect of Different Concentrations of Activated and Nonactivated PRC on hMSC Proliferation

**Figure 4:**
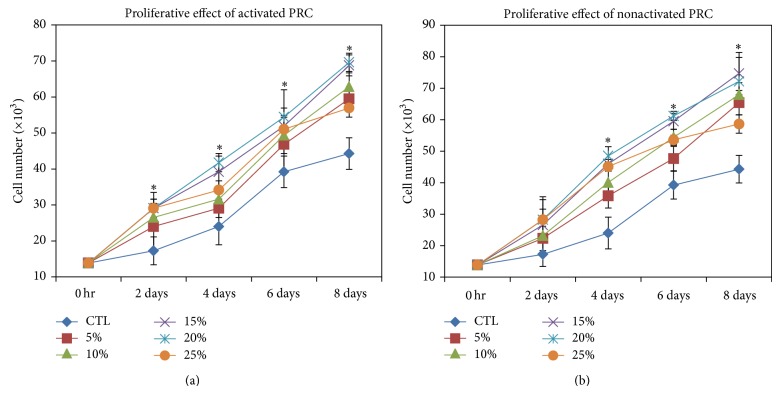
Effect of different concentrations of activated (a) and nonactivated PRC (b) on hMSC proliferation. Significant difference was observed between control and 15% activated PRC treated group at 2, 4, 6, and 8 days, while for the 15% nonactivated PRC group, the same was observed only from day 4 (^*^ *P* ≤ 0.05). There was no significant difference between 15% and 20% PRC treated groups.

The effects of activated and nonactivated PRC on hMSC proliferation are illustrated in Figure 4. Proliferation of hMSC gradually increased in both activated and nonactivated PRC groups in a dose-dependent manner with 25% showing a drop in cell proliferation. In the activated PRC group, cell proliferation was significantly increased at each time point compared to the control group (Figure 4(a)), while the same was observed only from day 4 in the nonactivated PRC treated cells (*P* ≤ 0.05) (Figure 4(b)). However, no significant differences in cell proliferation were observed between the activated and nonactivated PRC groups at day 8. In both the activated and nonactivated PRC groups, cell numbers from day 6 onwards were significantly higher in the culture supplemented with 15% and 20% PRC compared to other concentrations (*P* ≤ 0.05). However, as there was no significant difference in cell proliferation between the two concentrations at each time point (*P* > 0.05), 15% PRC concentration was used for the subsequent gene expression analysis.

### 3.6. Expression of Lineage-Specific Genes

**Figure 5:**
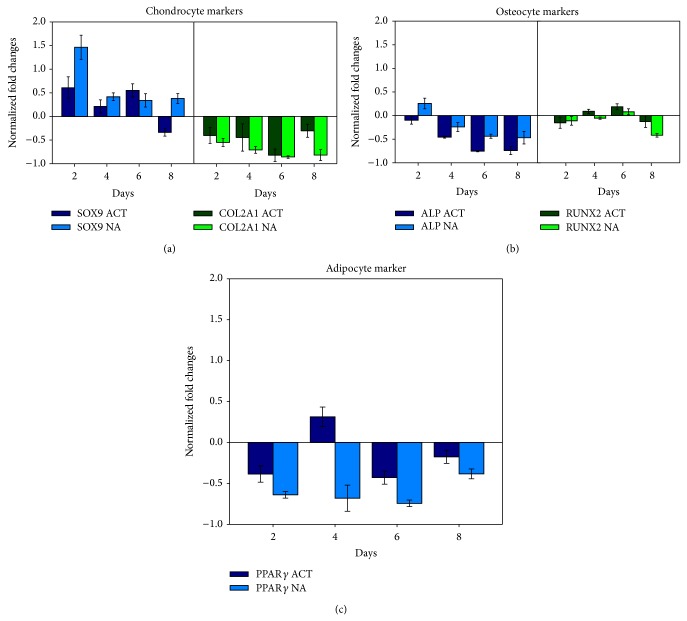
Gene expression levels of various markers specific to chondrocyte (a), osteocyte (b), and adipocyte (c) in hMSC cultured with 15% activated (ACT) and 15% nonactivated PRC (NA). SOX-9 expression was significantly increased at earlier time points in the nonactivated PRC group (^*^ *P* ≤ 0.05). Gene expression was measured at each time point using the QuantiGenePlex 2.0 assay kit.

Figure 5 shows the expression of various cell lineage markers in the hMSC culture treated with 15% PRC, expressed as a ratio normalized to the control. In general, although there were slight variation in the levels of cell markers at different time points, PRC treatment seemed to induce increased expression of SOX-9 (sex-determining region box 9). Despite the point-to-point variation in the gene expression levels, there were generally no significant difference in the pattern of expression of the various genes between the activated and nonactivated PRC groups. In groups treated with activated PRC, the expression of SOX-9 was upregulated from days 2 to 6 (Figure 5(a)). There was only a transient elevation in the level of the osteocyte marker, RUNX2 gene (Figure 5(b)), and PPAR-*γ* gene, an adipocyte marker (Figure 5(c)). All other genes (COL2A1, ALP) were found to be downregulated in the activated PRC group throughout the course of the experiment. In the nonactivated PRC treated groups on the other hand, SOX-9 genes were persistently upregulated throughout the experiment, with a more profound initial increase (1.5-fold) at day 2. A transient upregulation of the ALP and RUNX2 genes were observed at a few time points, but they remained downregulated on most days. COL2A1and PPAR-*γ* were downregulated within the time frame tested.

### 3.7. *In Vitro* Differentiation Assays and Histological Analysis

**Figure 6:**
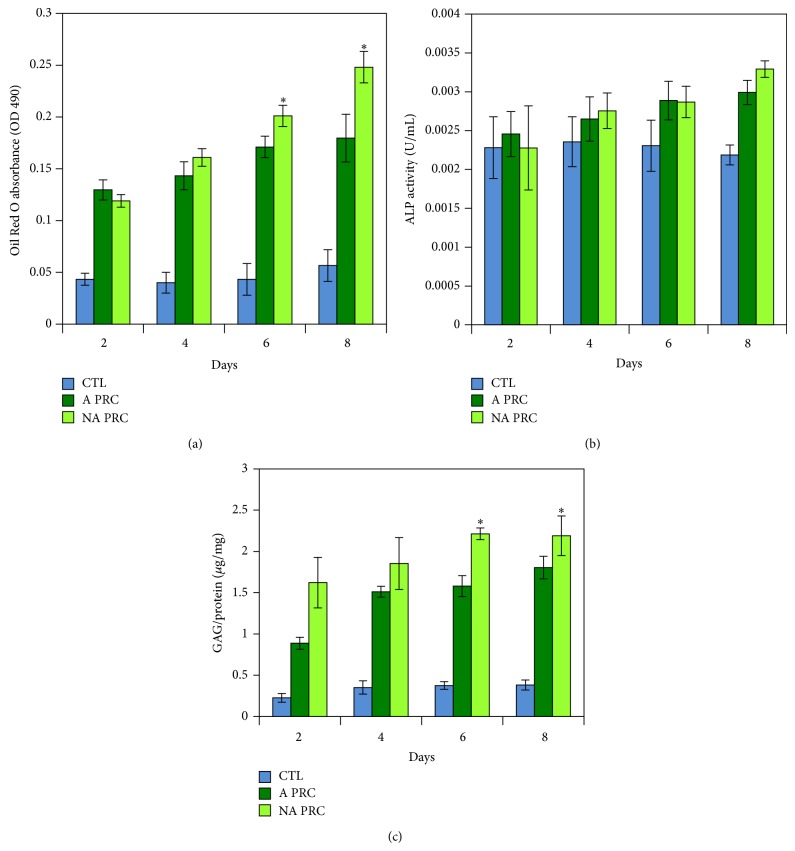
Differentiation assays. Adipogenesis assay shows significant increase in the nonactivated compared to activated PRC treated groups at days 6 and 8 (a). ALP activity showed no significant difference between activated and nonactivated groups (b). GAG was significantly higher in the nonactivated PRC treated groups than the activated PRC treated groups at days 6 and 8 (c). ^*^ *P* ≤ 0.05.

**Figure 7:**
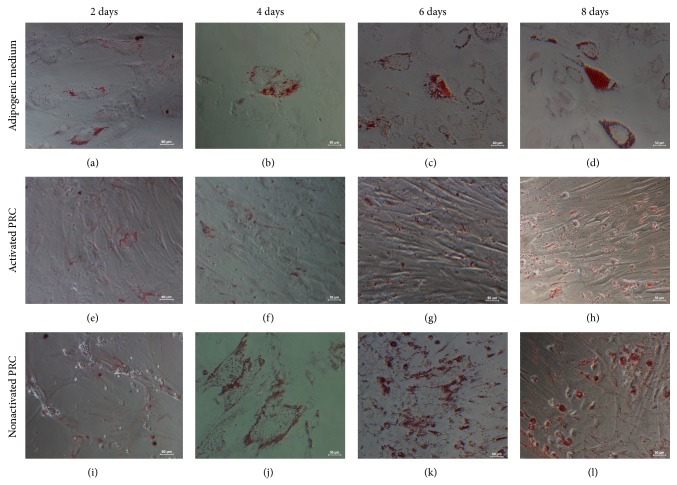
Cells stained with Oil Red O. Cells cultured in adipogenic medium (a)–(d), activated PRC (e)–(h), and nonactivated PRC (i)–(l). Cells cultured in PRC were fixed with formalin after each time point and were stained with Oil Red O. Cells cultured in adipogenic medium served as the positive control.

Significant level of adipogenesis was showed by positive Oil Red O staining (Figure 7) of lipid droplets present throughout the cytoplasm of the cells. The degree of adipogenesis was quantified using the adipogenesis assay (Figure 6(a)), which showed a significant increase in the intracellular lipid vacuoles in the cells treated with PRC compared to the control (*P* ≤ 0.05). In addition, there was significant difference in the groups treated with nonactivated PRC compared to activated PRC at days 6 and 8.

**Figure 8:**
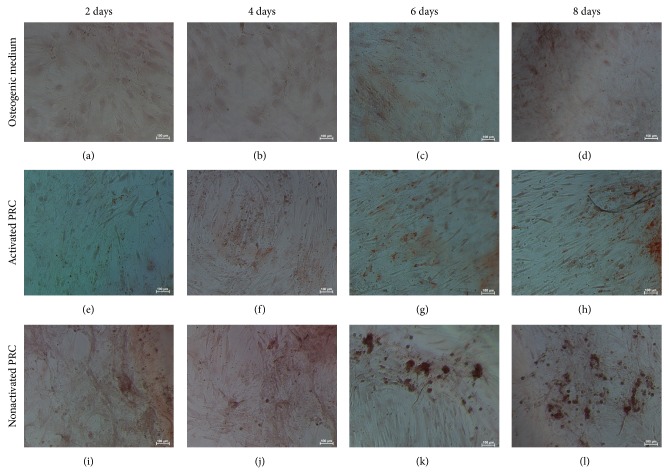
Cells stained with Alizarin Red. Cells cultured in osteogenic medium (a)–(d), activated PRC (e)–(h), and nonactivated PRC (i)–(l). Cells cultured in PRC were fixed with formalin after each time point and were stained with Alizarin Red. Cells cultured in osteogenic medium served as the positive control.

ALP activity (Figure 6(b)) showed a significant increase in PRC treated groups compared to the control from day 6 in the activated PRC treated groups and from day 4 in the nonactivated treated groups (*P* ≤ 0.05). However, there was no significant difference between the activated and the nonactivated groups. Cells showed matrix mineralization by day 8 as evidenced by Alizarin red staining (Figure 8).

**Figure 9:**
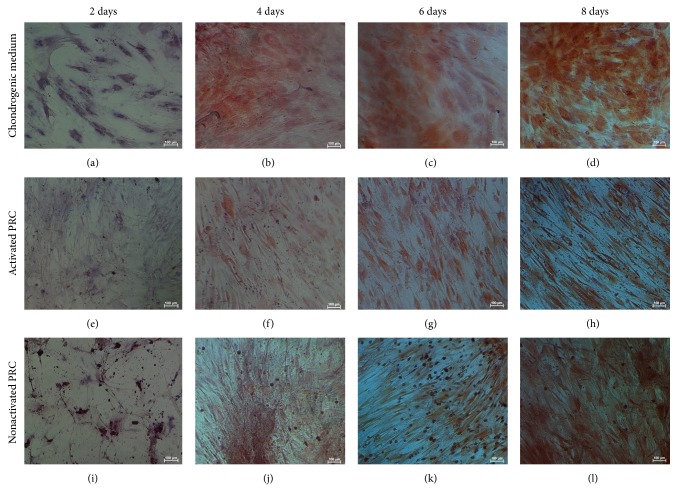
Cells stained with Safranin O. Cells cultured in chondrogenic medium (a)–(d), activated PRC (e)–(h), and nonactivated PRC (i)–(l). Cells cultured in PRC were fixed with formalin after each time point and were stained with Safranin O. Cells cultured in chondrogenic medium served as the positive control.

Accumulation of glycosaminoglycans (Figure 6(c)) in the extracellular matrix was significantly higher in the PRC treated groups compared to the control and there was significant increase in the nonactivated PRC groups only from day 6. The cells were also positively stained by Safranin O stain (Figure 9) which was prominent at day 8.

## 4. Discussion

The present study demonstrates a dose-dependent effect of PRC on hMSC proliferation until 20% concentration level. Maximal cell proliferation was observed in cultures treated with 15% and 20% PRC with no significant difference between them. In addition, gene expression and histological staining suggest that, within a short time frame of 8 days, hMSCs under the influence of PRC may have the propensity to differentiate into adipogenic and osteogenic lineages. It is interesting to note that these changes were evident as early as 2 days after treatment. The use of activated PRC provided no clear advantage over nonactivated PRC on the proliferation of cells. Thus, the findings of this study support the notion that, in view of its additive proliferative effect, PRC could potentially substitute the commercially available FBS or recombinant growth factors that are normally used for MSC expansion* in vitro*, as suggested by previous studies [16, 23].

Although many studies have investigated the role of PRP on proliferation of various native cell types including tenocyte [17, 24], osteoblast [13], and chondrocyte [25, 26], very few have focused on the use of PRP in the context of augmenting the proliferative and differentiation potential of human bone marrow-derived MSC. Our results show an inhibition in hMSC cell number with high platelet concentration (25%) similar to that observed by Mishra et al. [1] and Parsons et al. [27]. The drop in cell number with higher concentrations of platelets could be due to the presence of negative regulators such as thrombospondin, which is abundant in the *α*-granule of platelets. It is recognized that thrombospondin (TSP-1) proteins have the ability to inhibit endothelial cell proliferation and suppress angiogenesis [28]. Previous studies also show that the greater the concentration of TSP-1, the larger the decrease in cell proliferation [29].

A finding that is novel to this study is the fact that PRC can induce the differentiation of hMSC to different lineages as early as 8 days as opposed to previous studies which investigated its effect on differentiation of human MSCs to only one specific or predetermined lineage and for over a prolonged period [20, 27]. During the 8 days of treatment of hMSC with PRC, we showed that there is an increased propensity of hMSCs to differentiate to adipogenic and osteogenic lineages. Chondrogenic differentiation was not strongly observed, most likely owing to the fact that the cell cultures were done as monolayer instead of 3D culture, which is a prerequisite for chondrogenic differentiation to occur.

Platelets are known to contain several different growth factors stored in alpha granules that triggers a number of cellular functions [22]. The presence of various growth factors in platelets, which includes TGF-*β*1, PDGF AA, PDGF BB, PDGF AB, BMP, FGF, IGF, EGF, and VEGF, make PRC ideal for bone and soft tissue healing [22, 30]. These growth factors enhance cellular proliferation and also aid in the differentiation of cells, as shown in this study that PRC results in the significant osteogenic and adipogenic differentiation of hMSC evidenced by a transient increase in gene expression of ALP and PPAR-*γ* and also by the histological staining and biochemical assays. Although the present study did not investigate this in particular, we can assume that one of the growth factors involved is most likely transforming growth factor beta (TGF*β*), which promotes both osteogenic and chondrogenic differentiations [31]. The other growth factor that may be responsible for the observed differentiation to the osteogenic and adipogenic lineages would be PDGF (platelet derived growth factor) [32]. It has been suggested that these growth factors initiate the differentiation process by binding to the extracellular domain of a target growth factor receptor, which in turn activates the intracellular signal-transduction pathways [33]. TGF-*β* initiates the Smad signaling pathway by binding and activating the type II and type I receptor Ser/Thr kinases, which subsequently phosphorylate the Smad2/Smad3 effectors which form complexes with the common Smad and Smad4 and translocate into the nucleus regulating the transcription of the target genes like ACAN and SOX-9 [34]. Furthermore, TGF-*β* signaling also promotes osteoprogenitor proliferation, early differentiation, and commitment to the osteoblastic lineage through the Smad-dependent-BMP signaling and the cooperation between TGF-*β* and Wnt and the FGF signaling. Smad4 is a common Smad for both TGF-*β* and BMP signaling [35]. While PDGFs alone are not directly involved in the differentiation process, it indirectly regulates the bone regeneration by increasing the expression of angiogenic molecules, such as VEGF. PDGF-stimulated PI3K/Akt-mediated signaling enhances the TGF-*β*-induced osteogenic differentiation of hMSCs in a MEK/ERK-dependent manner. The combination of PDGF-activated PI3K/Akt and TGF-*β*-activated MEK mediates osteogenic differentiation which is important for optimizing the potential therapeutic use of hMSCs for bone formation [36]. In determining the probable mechanisms by which platelets are involved in promoting the proliferation and differentiation of MSC, it has become apparent from previous literatures that activation of platelets is necessary to release a myriad of growth factors that would induce both events simultaneously [15, 22]. It is unclear as to why the present study demonstrates that there are no differences between activated and nonactivated PRC, since the latter would not have resulted in the immediate release of the growth factors mentioned earlier. Although the proliferation of hMSCs treated with nonactivated PRC showed a delayed increase in number, but ultimately at 8 days there was no significant difference. It has been postulated that, in order to have the paracrine effect observed from platelets, a process of activation is required which involves the release of growth factors from the *α*-granules [22]. However, it is apparent from our results this is not the case and therefore warrants future investigation to be conducted.

This study has certain limitations that although were anticipated, were unavoidable. PRC was added only once at the beginning without any change of media throughout the experiment. Although medium replacement with PRC supplementation is preferentially done at a 3-day interval, we believe that frequent replacement of PRC in the growth medium might exaggerate the effects of PRC, as the cells may be receiving positive simulation from the introduction of fresh medium containing newly added PRC. Secondly, gene expression profiles of the cells cultured in the PRC-supplemented medium were analysed only up to 8 days. This duration may not be sufficient to track the entire differentiation process. Thirdly, differentiation towards the chondrogenic lineage was done in the monolayer although the optimal conditions would be a pellet or a 3D culture which would provide compaction and cell-cell contact needed for chondrogenesis. However, as our aim was to conduct a preliminary screen to indicate which lineage(s) hMSCs may be driven towards in the early stage of exposure to PRC, the short experimental duration and the use of monolayer cells deemed appropriate for its purpose. Nevertheless, further studies to follow up the differentiation process over longer time period in conditions optimal for the lineages are presently being conducted in our lab. We are also performing further studies to complement the gene expression data with their corresponding protein expression profiles to further strengthen our conclusion.

## 5. Conclusion

The present study demonstrated that the use of PRC enhanced proliferation of hMSC in a dose-dependent manner, albeit with certain limits, and an initiator of hMSC differentiation towards osteogenic and adipogenic lineages. SEM and flow cytometry results confirm that there was only minimal platelet activation during the preparation process. Preactivating PRC prior to its use did not appear to be necessary to achieve maximal proliferative effect. Although further work is warranted to support our conclusion through the use of more robust study design and tools, the data presented in this study does suggest a potential benefit of platelet rich concentrate to augment the proliferation and also the differentiation potential of hMSCs.
